# Predicting PD-L1 expression status in NSCLC using radiomic analysis of 18 F-FDG-PET/CT images

**DOI:** 10.1007/s00259-025-07453-2

**Published:** 2025-07-16

**Authors:** Fanni Júlia Kiss, Anna Izabell Járó, Domokos Máthé, Csaba Benedek, Vilmos Madaras, Andrea Manno-Kovács, Árpád László Bartha, Parasuraman Padmanabhan, Ramasamy Paulmurugan, Eszter Regős, Tamás Györke, Krisztián Szigeti

**Affiliations:** 1 Department of Nuclear Medicine, Medical Imaging Centre, Faculty of Medicine, Semmelweis University, Budapest, Hungary; 2 Department of Biophysics and Radiation Biology, Faculty of Medicine, Semmelweis University, Budapest, Hungary; 3 In Vivo Imaging Advanced Core Facility, Hungarian Center of Excellence for Molecular Medicine (HCEMM), Budapest, Hungary; 4 Institute of Nuclear Techniques, Budapest University of Technology and Economics, Budapest, Hungary; 5 HUN-REN Institute for Computer Science and Control (HUN-REN SZTAKI), Budapest, Hungary; 6 Faculty of Information Technology and Bionics, Pázmány Péter Catholic University, Budapest, Hungary; 7 Institute of Medical Microbiology, Faculty of Medicine, Semmelweis University, Budapest, Hungary; 8 Lee Kong Chian School of Medicine, Nanyang Technological University, Nanyang, Singapore; 9 Canary Center for Cancer Early Detection, Department of Radiology, Stanford University, Palo Alto, CA USA; 10 Department of Pathology, Forensic and Insurance Medicine, Faculty of Medicine, Semmelweis University, Budapest, Hungary

**Keywords:** Non-small cell lung cancer, PD-L1, Positron-emission tomography (PET), Computed tomography (CT), Radiomics, 18F-FDG-PET/CT

## Abstract

**Purpose:**

Lung cancer is the most prevalent malignancy globally, with prognosis and treatment largely influenced by histological and molecular analyses. Molecular features like PD-L1 positivity help identify patients suitable for immunotherapy. However, obtaining histological samples can be challenging and limited. Radiomic analysis of imaging data provides a non-invasive way to characterize tumor heterogeneity and its complex patterns, which may help predict PD-L1 expression. This study investigates the efficacy of radiomics in forecasting PD-L1 status in NSCLC patients using 18 F-FDG-PET/CT images.

**Methods:**

In this retrospective study, the primary staging 18 F-FDG-PET/CT scans of 105 patients with NSCLC of different phenotypes (72 ACC, 33 SCC, 64 PD-L1 positive, 41 PD-L1 negative) were analysed. Various segmentation techniques were employed. Radiomic features were obtained from the original and transformed images using the PyRadiomics package. Records were split into training and test sets in the ratio of 7:3. Feature reduction involved the Mann–Whitney U test, LASSO regression, and Spearman correlation analysis. A logistic regression model was developed using the selected features, and performance was assessed with ROC curve, AUC score, and other metrics.

**Results:**

The optimal model achieved an AUC of 0.783 (95% CI: 0.625, 942), with high accuracy (81.25%), sensitivity (90.00%), PPV (81.81%), and NPV (80.00%).

**Conclusion:**

Radiomic features derived from 18 F-FDG-PET/CT images can potentially differentiate between PD-L1 positive and negative NSCLC. Consequently, radiomics with multimodal imaging presents a promising non-invasive approach for selecting patients who may benefit from targeted immunotherapy.

## Introduction

This study demonstrates that using radiomics, it is possible to predict programmed death ligand 1 (PD-L1) expression status in non-small cell lung cancer (NSCLC) patients based on 2-deoxy-2-[18 F]-fluoroglucose-positron-emission tomography/computed tomography (18 F-FDG-PET/CT) images. This non-invasive approach can help identify patients who may benefit from immune checkpoint inhibitor (ICI) therapy [1]. By utilizing this method, we aim to support clinical decision-making and improve patient outcomes.

Malignancies are marked by significant intra-tumoral heterogeneity; this presents challenges for the clinical management of cancer patients. Spatial heterogeneity refers to the variation in the genetic and phenotypic characteristics of tumor cells within different regions of the same tumor or even between primary and metastatic sites. Temporal heterogeneity involves changes in these characteristics over time, often due to treatment and other environmental factors [2–4]. 

Medical imaging plays a crucial role in understanding the biology of malignancies. Firstly, unlike tissue sampling for histology, it is a non-invasive, in vivo diagnostic tool. Secondly, medical imaging enables the assessment of tumor system dynamics over an extended period by repeating the examinations. Thirdly, it provides insights at different depth levels to characterize various cancer processes, such as molecular, functional, and anatomic imaging, providing an opportunity to examine intra-tumoral heterogeneity. Lastly, unlike biopsies, which provide limited information from a single tissue sample, tomographic techniques such as PET, CT, and magnetic resonance imaging (MRI) offer a comprehensive, three-dimensional picture of the entire cancerous region, including nearby structures and potential metastases [2]. 

Radiomics exploits these advantages by using medical images to extract quantitative information that is difficult for human eyes to recognize or quantify. The primary hypothesis of radiomics is that the extracted features should correlate with gene expression pattern for therapeutically valuable markers, providing the basis for digital biopsy [4–6]. 

### NSCLC and immunotherapy

According to the latest global cancer statistics, the most prevalent form of malignancy worldwide is lung cancer, which is responsible for the highest number of cancer-related deaths [7]. Early and accurate diagnosis and staging are crucial to improve patient outcomes. In over 50% of cases, curative surgery is not an option [8]. Tissue sampling is essential as the future treatment depends on the histological type of the tumor. NSCLC represents ∼ 85% of diagnosed lung cancer cases and includes various subtypes, such as adenocarcinoma (ACC) and squamous cell carcinoma (SCC).

**Fig. 1:**
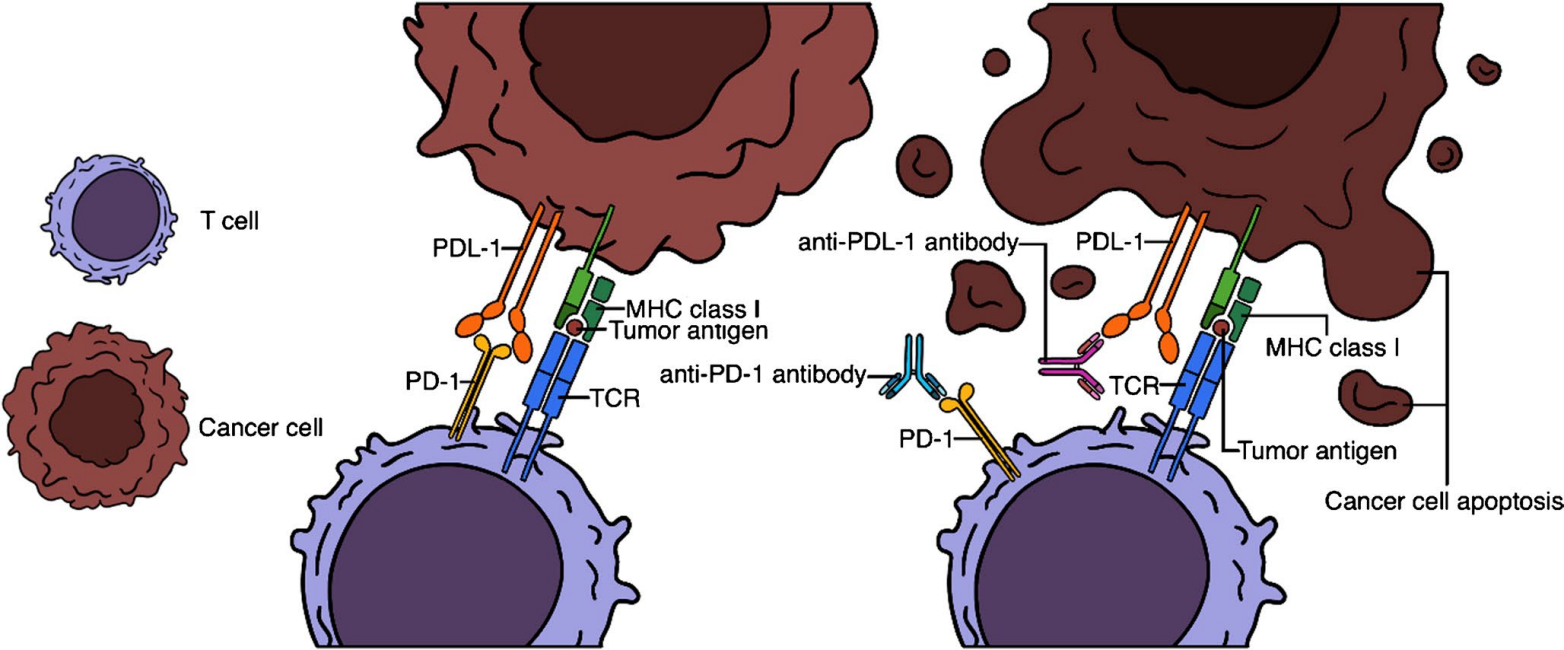
Pictorial illustration showing the mechanism of action by ICIs in cancer immunotherapy. TCR: T-cell receptor, MHC: major histocompatibility complex

The immune system has several checkpoints to prevent the destruction of normal cells, but malignancies can manipulate these to establish tolerance. Among the checkpoints, programmed death receptor 1 (PD-1) has received particular interest recently due to its high potential in treating many cancers. Figure 1. shows the interaction between PD-1 and PD-L1 and PD-L2 proteins. Currently, there are antibodies approved for targeting PD-1 and PD-L1. According to the latest version of the National Comprehensive Cancer Network (NCCN) Clinical Practice Guidelines in Oncology, depending on the PD-L1 expression status, they can be prescribed as first-line or subsequent treatment with or without chemoradiotherapy or as part of neoadjuvant and adjuvant therapy for squamous and non-squamous NSCLC. They may be suitable for inoperative or potentially operative cases, and compared to platinum-based chemotherapy, they have fewer, usually mild side effects [9]. Currently, PD-L1 expression is the most reliable biomarker for identifying patients who can benefit from PD-1 or PD-L1 inhibitor therapy. Immunohistochemistry (IHC) remains the gold standard for assessing PD-L1 expression status. Still, there has been particular interest recently in quantifying PD-1/PD-L1 expression and its heterogeneity in vivo with ^89^Zr-labeled anti-PD-1/PD-L1 monoclonal antibodies [1, 8, 10]. In lung cancers, PD-L1 expression status is routinely assessed only in ACC and SCC.

Histological sampling is often time-consuming, complicated, or unsuccessful (e.g., due to extensive emphysema or the undesirable location of the tumor). Complications can also occur, such as pneumothorax, bleeding, and infections. In some institutions, placing patients after the procedure presents a challenge. The performance status of some patients may not allow sampling at all. Additionally, the pathological analysis of the sample is usually a time-consuming process. Also, PD-L1 expression status can be altered by the heterogeneity of the tumor and by treatment [11]. This highlights the need for new non-invasive techniques, such as radiomic analysis of imaging data. Radiomics has potential clinical value in situations where a biopsy is not possible. Additionally, it can be a viable alternative for cases requiring repeated monitoring or reassessment of treatment effects, as it offers more detailed information about tissue changes than traditional methods. In instances where multiple biopsies would be difficult or impractical, radiomic analysis can help determine whether tissue sampling is necessary. Furthermore, radiomics lays the groundwork for further research, which may uncover stronger connections to biological markers.

### 18 F-FDG-PET/CT imaging in treatment-naive NSCLC

Per the NCCN recommendations, all patients with suspected NSCLC should undergo a PET/CT scan, which provides information on the metabolic activity of the cancer processes. 18 F-FDG is the most commonly used PET radiotracer in the management of NSCLC. 18 F-FDG uptake varies among different lung histological subtypes: squamous cell carcinoma typically shows higher 18 F-FDG avidity than adenocarcinoma [12]. 

18 F-FDG-PET/CT can efficiently differentiate between benign and malignant lesions. A maximum standardized uptake value (SUV_max_) exceeding the mean mediastinal blood pool activity or 2.5 is suggestive of malignancy. Due to limited spatial resolution, 18 F-FDG-PET/CT is not recommended for lesions smaller than 8 mm. 18 F-FDG-PET/CT should also be avoided in the characterization of subsolid opacities to prevent false-negative (e.g., atypical adenomatous hyperplasia, carcinoma in situ, minimally invasive carcinoma) and false-positive results (e.g., inflammation or infection) [13]. Small lesions smaller than 15 mm in the lower lobes may lead to inaccuracies due to artifacts caused by breathing motion; thus, respiratory gating is strongly recommended in these scenarios [14]. 

### The role of radiomics in determining PD-L1 expression status

One of the most promising advancements in oncologic imaging is the field of radiomics. Radiomics involves extracting features from medical images and analysing the acquired complex data using advanced machine learning and statistical methods. Radiomic features may not only characterize tumor heterogeneity but also provide additional complementary information to other available sources: demographics, pathology, blood biomarkers, and genomics, which could play essential roles in diagnostics, patient-specific treatment monitoring, and patient prognosis [4, 5]. 

## Materials and methods

### Patient selection, dataset

In this study, 18 F-FDG-PET/CT scans of a total of 148 patients collected from March 2017 to March 2021 were analysed retrospectively. The necessary ethical approvals were obtained from the Hungarian Medical Research Council and the Scientific and Research Ethics Committee review board (BM/24985-1/2024, retrospectively registered). We used the following parameters as the inclusion criteria for the samples: (1) histologically proven NSCLC with available molecular features; (2) primary staging PET/CT, as treatment can alter PD-L1 expression status; (3) the size of the primary tumor had to be at least 8 mm; (4) the CT appearance of the primary tumor had to be solid; and (5) the activity of the primary tumor had to exceed the mean activity of the mediastinal blood pool, measured in the descending aorta. Upon inclusion in the study, patients were anonymized. We used the following exclusion criteria: (1) unknown PD-L1 expression status (*n* = 22); (2) incomplete or incorrect image set (*n* = 12); and (3) peritumoral atelectasis (*n* = 9). After implementing the exclusion criteria, we had a dataset of 105 patients for the radiomics analysis.

**Fig. 2:**
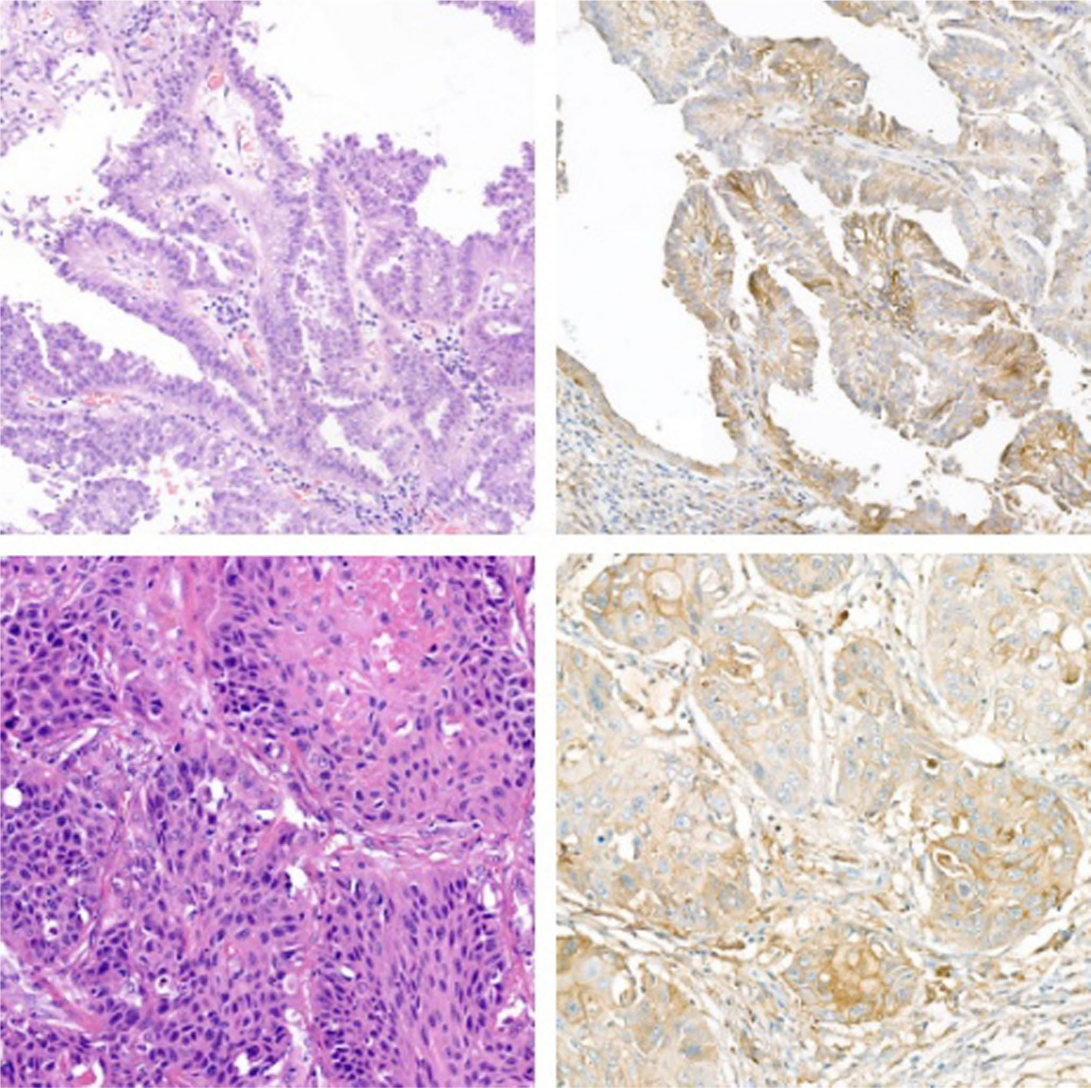
NSCLC histological samples. Top: ACC, bottom: SCC. Left: hematoxylin and eosin staining, right: IHC. The ACC shows approximately 80% PD-L1 positivity, while the SCC shows 100% PD-L1 positivity

Tissue samples were obtained by biopsy or surgical resection, as studies have shown no significant difference in the feasibility of PD-L1 IHC on biopsy specimens compared with the tissue samples obtained by surgical resection [15]. The PD-L1 expression status of each patient was determined by an experienced pathologist by IHC (Fig. 2.). PD-L1 expression level ≥ 1% was considered positive (64 patients), and < 1% was considered negative (41 patients). Table 1. summarizes the available demographic and clinical characteristics of patients according to the PD-L1 expression status. Mainly, elderly female and male patients were included from all stages to reproduce routine clinical conditions reliably. Most of the patients were former or active smokers.

**Table 1:** The demographic and clinical characteristics of patients with NSCLC and the NSCLC phenotype according to the PD-L1 expression status

Data	PD-L1 positive	PD-L1 negative	Summary
Age, mean (years)	67.6	67.3	
Gender, N (%) Female Male	28 (34.3%) 36 (26.7%)	21 (19%) 20 (20%)	49 (46.7%) 56 (53.3%)
Histological type, N (%) ACC SCC	39 (37.1%) 25 (23.8%)	33 (31.4%) 8 (7.6%)	72 (68.6%) 33 (31.4%)
TNM stage, N I IA IB II IIA IIB III IIIA IIIB IIIC IV	5 4 1 10 3 7 27 17 7 3 22	5 3 2 7 1 6 11 6 4 1 18	10 7 3 17 4 13 38 23 11 4 40
Smoking history, N Never Ex-smoker Smoker	11 18 35	9 16 16	20 34 51

### Patient preparation, 18 F-FDG-PET/CT acquisition protocol

18 F-FDG-PET/CT scans were obtained at the Department of Nuclear Medicine, Medical Imaging Centre, Semmelweis University, based on the latest version of the European Association of Nuclear Medicine (EANM) procedure guidelines [16]. Before the examination, a minimum fasting of 6 h was required, considering that the blood glucose concentration should not exceed 11.1 mmol/L. For PET acquisition, we used a pharmaceutical grade of 18 F-FDG. 2.5 MBq/kg (or 3 MBq/kg for individuals with a body mass index [BMI] greater than 30) 18 F-FDG was administered intravenously. The uptake time was between 55 and 65 min. The scans were performed on the GE Discovery IQ 5 PET/CT system from the base of the skull to the mid-thigh with elevated arms. The PET/CT acquisition protocol included a topogram, a low-dose non-contrast CT for attenuation correction and anatomical correlation, a PET, and a chest CT at full inspiration. Respiratory gating was utilized for small lung lesions (less than 15 mm) in the lower lobes. The CT acquisition parameters were 120 kV (or 140 kV for those with a high BMI) and 180 mAs. The PET acquisition time for each bed position was 2 min (or 2.5–3 min for those with a high BMI). Following iterative reconstruction, the images had a voxel size of 512 × 512 × 319 for CT and 256 × 256 × 319 for PET, and spacing is 0.977 mm, 0.977 mm, 3.260 mm for CT and 2.734 mm, 2.734 mm, 3.260 mm for PET.

### Segmentation

**Fig. 3:**
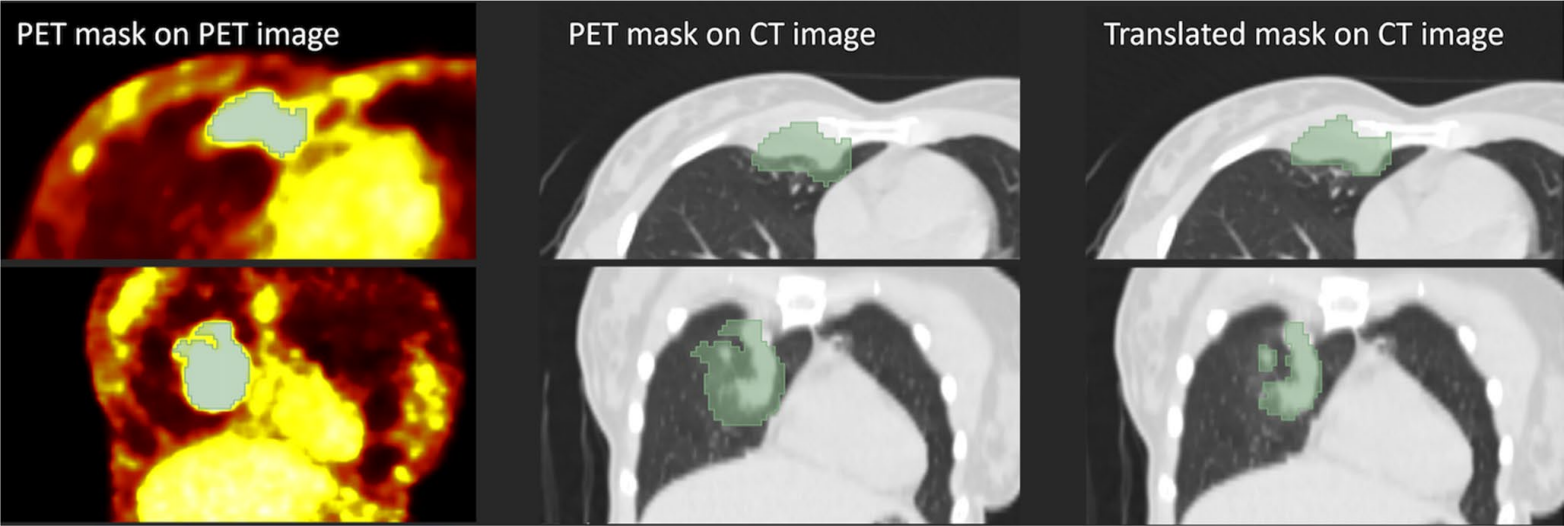
Segmentation of the PET and the CT images. The first row shows axial, and the second shows coronal slices. Left: PET image with the original mask. Middle: CT image with the upsampled PET mask (Case 1). The tumor is not adequately covered, potentially affecting the extracted radiomics features from the segmented area. Right: CT image with the upsampled and translated mask (Case 2) shows a better fit to the tumor after the transformation

For all the patients, segmentation was done manually on the Bayesian penalized likelihood (PL) reconstruction algorithm (Q.Clear) for PET images by a nuclear medicine physician with more than five years of experience in using the latest available version of InterView™ FUSION software from Mediso Medical Imaging Systems Ltd. The region of interest (ROI) that covered the entire primary tumor was delineated by an isocontour, a three-dimensional (3D) drawing tool based on adaptive thresholding associated with a region-growing approach proposed by the software. The maximum and the peak standardized uptake value (SUV_max_ and SUV_peak_) of the primary tumor were calculated automatically on the Q. Clear and Ordered Subsets Expectation Maximization (OSEM) reconstruction PET images. Since respiratory gating was not performed in all cases, segmentation and SUV calculation were done on the non-respiratory gated images for exact comparability. Due to differences in the timing, PVE of CT and PET acquisitions, and respiratory motion artifacts in PET images, the tumorous area of the lung did not always overlap on the fused images. Hence, the translation of the PET ROI onto the CT image may have been slightly incorrect. This phenomenon can be seen in Fig. 3. in a representative case.

As PET and CT do not have the same spatial resolution, all masks have been resampled. The sitkNearestNeighbor interpolation algorithm was used to resample the masks to preserve the label values. For image interpolation, the sitkBSpline algorithm was applied. The tumor segmentation from the PET image was either directly transformed to the CT image (Case 1) or manually defined using translation vectors to correct for misalignment between the PET and CT images (Case 2). The manual correction involved using 3D Slicer™ version 5.2.1 to qualitatively assess and adjust the alignment across three cross-sections of the 3D images, applying a transformation vector to the PET mask where necessary.

### Feature extraction

Radiomics features were extracted from both PET and CT images using the PyRadiomics™ Python package directly and as an extension of 3D Slicer. The derived features include morphological, intensity-based, and texture parameters. The collection of the feature names can be found in [17].

In addition to the original image, wavelet, and Laplacian of Gaussian (LoG) transforms were conducted on the CT images and used for further feature extraction. In addition to the original image, wavelet and LoG transforms were conducted on the CT images using the default combinations (LLL, LLH, LHL, LHH, HLL, HLH, HHL, HHH) for the wavelet transform, and sigma values of 1, 2, 3, and 4 mm for the LoG transform. Normalization and resampling were performed for 1 × 1 × 1 mm [3] in the X, Y, and Z axes for both PET and CT feature extraction. Bin width of 25 was used for both images.

### Feature selection, model construction, and evaluation

The following analysis was performed in Python using the Scikit-learn, SciPy, and Seaborn packages. Mann–Whitney U test, LASSO regression, and Spearman’s rank-order correlation analysis were used to construct and evaluate the model.

## Results

### SUV

The comparison between SUV_max_ and SUV_peak_ values (Table 2.) was performed using Mann–Whitney U test. The results revealed significant differences for PD-L1 positive and negative patients, with corresponding p-values of 0.00039 and 0.00044, respectively.

**Table 2:** The median values of SUV_max_ and SUV_peak_, according to the PD-L1 expression status

Data	PD-L1 positive	PD-L1 negative
SUV_max_, median (IQR)	15.3 (13.4, 20.6)	12.3 (8.75, 16.1)
SUV_peak_, median (IQR)	12.8 (10.55, 16.5)	9.2 (7.43, 13.18)

### Feature selection

The dataset was divided randomly into training and test groups in the ratio of 7:3; the splitting was stratified by the label variables (PD-L1 expression status) to maintain the same ratio of PD-L1 negative and positive patients in both cohorts [18–20]. For further analysis steps starting from feature selection, only the training set is used to avoid data leakage, and the test set is used exclusively for model evaluation.

**Fig. 4:**
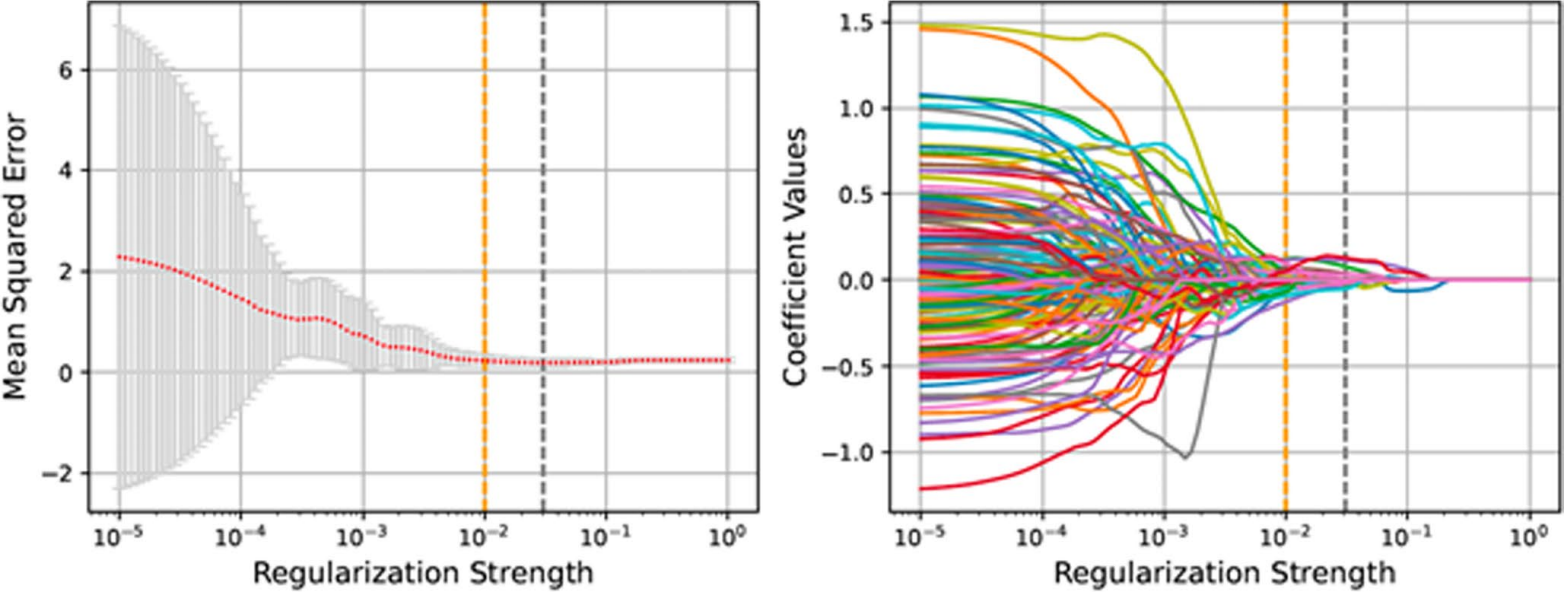
Feature reduction with the LASSO algorithm. Left: selecting the optimal α parameter (regularization strength) with ten-fold cross-validation. The gray, vertical dashed line represents the optimal α obtained from cross-validation, and the orange line represents the chosen α used in further analysis. Right: penalty diagram of the radiomics feature coefficients during training of the LASSO model with different α-s. This shows how the coefficients shrink to zero depending on the regularization strength

Regarding the huge number of extracted features, dimension reduction is needed to avoid overfitting when training the model. The Mann–Whitney U test identified redundant features upon checking differences in distribution between PD-L1 negative and positive groups. Features with p-values above 0.05 were excluded from the analysis. The reduced dataset was standardized prior to regression analysis in both training and validation sets. The LASSO algorithm further narrowed down feature numbers, distinguishing between significant and non-significant values. Ten-fold cross-validation determined the optimal regularization hyperparameter with an α of 0.03. The applied regularization strength was α = 0.01, close to optimal and within one standard error (Fig. 4.).

**Fig. 5:**
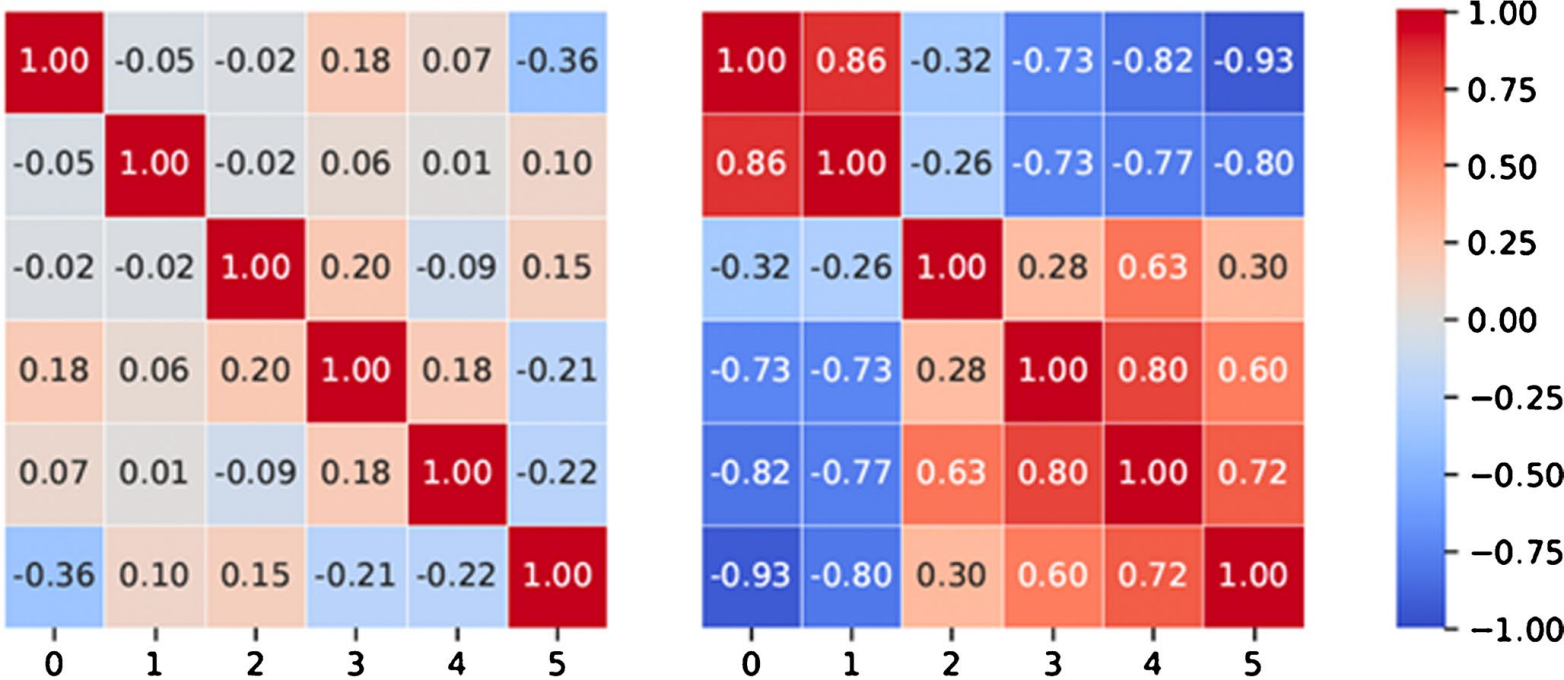
Correlation matrices for the selected features. Pairs with Spearman correlation coefficient higher than 0.9 in absolute values were identified, and only those features with a higher correlation with the target variable PD-L1 status were kept. Left: Case 1, Right: Case 2

Lastly, Spearman’s rank-order correlation analysis was performed to determine the strength of the monotonic relationships between selected features. Pairs with correlation coefficient higher than 0.9 in absolute values were filtered out, and only those with higher correlation with the target variable PD-L1 status were kept (Fig. 5.).

### Model construction and evaluation

After feature selection, a linear logistic regression classifier model was constructed using the remaining features. The feature selection process reduced the number of independent variables to one order of magnitude smaller than the training dataset, thereby enhancing the robustness of the model and reducing the likelihood of overfitting.

The selected features encompass various metrics derived from both PET and CT images. For instance, in Case 1, features such as the wavelet-LHH glcm SumEntropy and wavelet-HLH glszm HighGrayLevelZoneEmphasis highlighted the importance of texture analysis, which captures the spatial arrangements of pixel intensities within the tumor. Including features like the wavelet-HHH firstorder Range and the original firstorder Median PET further emphasized the significance of intensity variations in tumor characterization. The log-sigma-3-0-mm-3D ngtdm Complexity feature provided additional insights into the complexity of the texture of the tumor. In Case 2, features such as wavelet-LHL glszm Zone% and original firstorder TotalEnergy illustrated the capability of the model to assess the distribution and overall intensity of tumor uptake, which are critical for understanding the metabolic activity. Meanwhile, the wavelet-LHL gldm DependenceNonUniformityNormalized feature reflected the uniformity of the image intensities within the selected region. In contrast, the presence of the log-sigma-3-0-mm-3D ngtdm Busyness feature suggested that texture characteristics were vital in distinguishing between positive and negative PD-L1 expression status.

We run the entire extraction process for PET images with bin width of 1 and 25, but found an average deviation less than 1% in the values for the above-mentioned features. Accordingly, and based on the recommendations found in the literature, we reported our results corresponding to the bin width of 25 value.

**Fig. 6:**
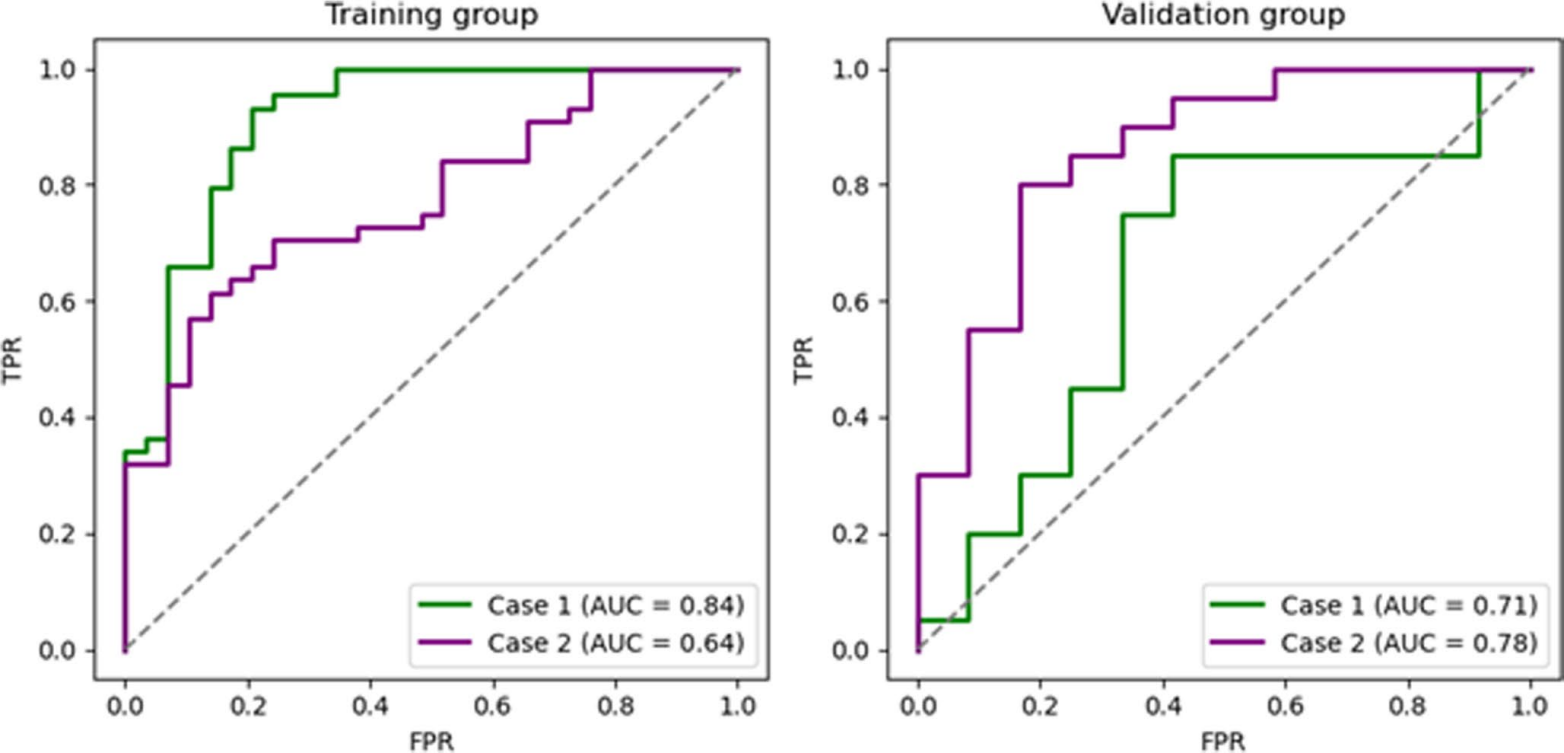
ROC curves with corresponding AUC scores in different cases

To assess model performance, receiver operating characteristics (ROC) curves were plotted for both the training and test sets for each case. The analysis included key performance metrics such as the area under the curve (AUC) score, accuracy, sensitivity, specificity, and positive and negative predictive values (PPV and NPV). These metrics provided a comprehensive assessment of the predictive capabilities of the model. Figure 6. illustrates the ROC curves along with their corresponding AUC scores for the various cases examined, providing a visual representation of the classification performance of the model.

Table 3. presents the coefficients of the selected features from the logistic regression models for predicting PD-L1 expression status. This table details the name, transformation type (original, wavelet, or LoG transformation), modality (CT or PET) of each feature, and its associated coefficient, which indicates the strength and direction of the association of the features with the prediction outcome. The intercept values for each model are also included, highlighting the baseline log-odds for predicting PD-L1 status.

**Table 3:** Coefficients of selected features from the logistic regression models for predicting PD-L1 expression status. Each case lists the transformation type (original, wavelet, or log transformation), name of the feature, modality, and the corresponding coefficient, indicating the strength and direction of the association with the prediction outcome. The intercept values for each model are also included

Case	Feature name or intercept	Modality	Coefficient
1	wavelet - LHH glcm SumEntropy	CT	0.9904
	wavelet - LHH glrlm HighGrayLevelRunEmphasis	CT	−0.7964
	wavelet - HHH firstorder Range	CT	−1.0039
	log - sigma-3-0-mm-3D ngtdm Complexity	CT	−0.8699
	wavelet - HLH glszm HighGrayLevelZoneEmphasis	CT	0.5315
	original - firstorder Median	PET	0.9514
	intercept		0.6487
2	wavelet - LHL glszm Zone%	CT	0.9961
	original - firstorder InterquartileRange	PET	0.0688
	wavelet - LHL gldm DependenceNonUniformityNormalized	CT	0.5545
	original - firstorder TotalEnergy	PET	0.8494
	log - sigma-3-0-mm-3D ngtdm Busyness	CT	−0.6209
	intercept		0.5841

In Table 4., we provide a comprehensive overview of the performance metrics, including confidence intervals for the AUC values of the logistic regression models, assessing their effectiveness in predicting PD-L1 expression status across both training and test datasets for the two cases. The best-performing model achieved the highest AUC of 0.783 (95% CI: 0.625, 0.942) on the validation group, together with a high accuracy of 81.25%, sensitivity of 90.00%, specificity of 66.67%, PPV of 81.81%, and NPV of 80.00%.

**Table 4:** Performance metrics of the logistic regression models for predicting PD-L1 expression status in both training and test datasets across two cases. Metrics include AUC, accuracy, sensitivity, specificity, PPV, and NPV, providing a comprehensive assessment of model efficacy. AUC values are presented alongside their respective confidence intervals

Metric	Case 1		Case 2	
	*Test*	*Training*	*Test*	*Training*
AUC (95% CI)	0.708 (0.528, 0.889)	0.840 (0.750, 0.929)	0.783 (0.625, 0.942)	0.639 (0.512, 0.766)
Accuracy (%)	71.87	84.93	81.25	67.12
Sensitivity (%)	75.00	88.63	90.00	79.55
Specificity (%)	66.67	79.31	66.67	48.28
PPV (%)	78.94	86.67	81.81	70.00
NPV (%)	61.54	82.14	80.00	60.87

This evaluation underscores the potential of the logistic regression model for accurately predicting the PD-L1 expression status, ultimately contributing valuable insights for clinical decision-making in the context of personalized therapy for NSCLC patients. The strategic selection of features and robust evaluation methodologies collectively enhance the reliability and interpretability of the predictive models.

## Discussion

The overall results of our analysis suggest that PD-L1 positive tumors show significantly higher glucose metabolism. The molecular mechanism between PD-L1 expression and glucose metabolism has not been fully revealed. PD-L1 expression is considered to play a role in the protein kinase B (Akt)/mammalian target of rapamycin (mTOR) signaling pathway, which promotes the glucose metabolism of the tumor through the synthesis of glycolytic enzymes [21]. Further, PD-L1 is considered to be a direct target of hypoxia-induced factor-1α (HIF-1α), which can also increase glycolysis in tumor cells by regulating the expression of glucose transporter 1 (GLUT-1) [22]. PD-L1 expression (and, therefore, also glucose metabolism) is known to be heterogeneous within the tumor. Despite its widespread use, it is important to note that SUV is a semi-quantitative measurement tool that does not capture intra-tumoral heterogeneity in metabolism. Furthermore, it can be influenced by many equipment, physical, and biological variables, which are difficult to define and regulate. As a result, its predictive value remains weak [13]. 

Five features were selected using the methods detailed above. The different mathematical approach to feature types ensures that multiple spatial features suitable for tumor characterization are considered, and the correlation of the selected parameters helps to reduce multicollinearity. The precise identification of actual PD-L1-positive cases is crucial, as PD-L1 inhibitors are used as first-line monotherapy in non-small cell lung cancer (NSCLC) for patients with at least 50% PD-L1 expression.

In recent years, several publications have addressed similar topics, using radiomics or artificial intelligence-based methods, each with varying approaches to improve diagnostic accuracy. We believe that to build confidence in radiomics and facilitate its integration into clinical practice, it is essential not only to develop novel methods but also to rigorously apply and validate existing approaches across diverse patient populations. Growing evidence on this subject contributes to the overall credibility of radiomics in biomarker prediction and may ultimately support its routine adoption in oncology. With our study, we aim to contribute to this evolving field and help pave the way toward the clinical implementation of radiomics. To our knowledge, the performance value of radiomics in predicting PD-L1 expression status has rarely been investigated (Table 5.), particularly in the European context where such studies are nearly absent. Jiang et al. [23] published the first research on the subject in which radiomic analysis was performed on a relatively large dataset. They found that the PET and the combined PET/CT features were less related to PD-L1 expression than the CT features. This finding is contrary to the results obtained in our study. However, this study is characterized by employing low-resolution PET images and suboptimal CT segmentation, potentially influencing the results. They delineated the tumors on the full inspiration CT images and were then transformed on the free-breathing PET images. Additionally, there is only one approved companion diagnostic to select patients for PD-1/PD-L1 inhibitor therapy, and in some cases, they use different antibody clones for IHC. Finally, they mainly consider surgical specimens from primary tumors, which limits the translatability of this approach to clinical practice, as the histopathological status often relies on cytological or histological samples, even those taken from metastatic sites. Li et al. [18] and Zhao et al. [19] reached another conclusion by analysing a relatively large dataset. They established a clinical and a radiomic model incorporating the demographic and clinical characteristics with the radiomic signature into a combined model. They showed that fusing CT and PET features improves the ability of the model to predict the PD-L1 expression level, which is consistent with our findings. Similarly, Wang et al. [24] successfully distinguished PD-L1 positive tumors from negative ones by using PET/CT radiomics. They found that the PET-based radiomic model performed better than the CT-based one. This result may be due to suboptimal CT segmentation. Dilatation was performed after manual segmentation, which may have caused the ROI not to follow the tumor contour precisely. Li et al. [20] constructed a PET/CT radiomics, a deep learning, and a fused model. They found that the fused model outperformed both models, suggesting that adding deep learning features can offer several advantages for assessing PD-L1 expression status. However, further research is required as this study was conducted on a small database. Additionally, the description of their segmentation method is not detailed enough. It is unclear which CT series was used as a reference for PET segmentation, and it is also unclear how the differences between PET and CT modalities were considered. The convolutional neural network may have struggled to extract deep learning features effectively because it only utilized one slice of the image, which contained the tumor in its greatest diameter, potentially excluding crucial information.

**Table 5:** Summary table of results of articles published on this topic

Authors	Journal	Publ. date	Num. of patients	Results
Mengmeng Jiang	Acad Radiol	02/01/2020	399	AUC = 0.71, 0,86
Jihui Li	Front Oncol	12/16/2021	255	AUC = 0.762, 0.814
Jianyuan Zhou	Front Oncol	11/17/2021	103	AUC = 0.794
Y.B. Wang	Clin Radiol	06/21/2023	394	AUC = 0.813
Bo Li	EJRO	01/19/2024	136	AUC = 0.829
Xiaoqian Zhao	EJNMMI	01/22/2023	334	AUC = 0.761, 0.769

Recent studies have shown that the blockade of PD-1/PD-L1 is unlikely to achieve any antitumor efficacy without preexisting CD8 + tumor-infiltrating lymphocytes (TILs). Tumors with tumor microenvironment type I (TMTI-I), such as those with high PD-L1 expression and the presence of CD8 + TILs, are more likely to respond positively to anti-PD-1/PD-L1 therapy. Zhou et al. [25] assessed the tumor immune microenvironment with 18 F-FDG-PET/CT radiomics in a relatively small dataset to predict the immune phenotype. They constructed a clinical, a radiomic, and a combined model. They only found PET and CT features that showed moderate ability to predict PD-L1 expression status.

The primary limitation of this research is its limited sample size. Additionally, it was a single-center study; images were performed on only one PET/CT scanner, and the tissue samples were most often taken from the primary tumor. Although these concerns may limit the generalizability of our findings, this study was conceived as a proof-of-principle investigation to demonstrate the potential of radiomics-based prediction of PD-L1 expression status for aiding clinical decision-making in oncology. Notably, recent works in the field have reported similar sample sizes, underscoring the relevance and feasibility of studies at this scale. Importantly, our results are comparable to those of the cited studies despite being derived from a distinct population. This supports the applicability of our approach across different clinical settings. Future work will focus on expanding the patient cohort, conducting multi-center data from different PET/CT scanners, and adopting bioptic materials from various sources to further validate and strengthen the robustness of our predictive model. Another area for improvement is the quality of segmentation. The analysis of tumor histograms regarding the manual masks shows excellent promise for enhanced predictive performance. However, other segmentation methods should also be considered, as manual segmentation has poor reproducibility and is extremely time-consuming. The precise manual delineation of tumors can present significant challenges, mainly when dealing with tumors that have spiculated margins, which are not uncommon in clinical practice. In this study, we aimed to segment the tumors as accurately as possible; however, achieving precise delineation of all spiculations proved nearly impossible. While spiculated margins can indicate malignancy, they are generally not factored into the measurement of tumor diameter. In radiomics, however, these margins may be particularly important, revealing a notable discrepancy between clinical assessments and radiomic evaluations.

Possible research directions include predicting not only PD-L1 expression status but also percentage levels and other molecular pathological features. Comparing different radionics software for feature extraction from the same dataset could help achieve reproducible results and standardized features. In addition, various deep learning methods could be incorporated to determine the most effective modeling approach.

## Conclusion

This study explored the potential of using radiomic features extracted from 18 F-FDG-PET/CT images to predict PD-L1 expression status in NSCLC patients. Our goals included finding the best segmentation scheme for optimal and precise feature calculation, evaluating the impact of image transformation on feature extraction, performing a comprehensive radiomic analysis with accurate feature selection, and developing a robust binary classification model for PD-L1 expression status.

In conclusion, the above findings significantly help create precise and robust PET/CT radiomic models. These models offer a promising way to predict PD-L1 expression status in NSCLC patients, providing a non-invasive method for making personalized therapy decisions, especially in challenging situations. Radiomics has the potential to change clinical protocols by offering a convenient and comprehensive alternative to histological and molecular analysis. The ability to improve patient outcomes through specific treatment makes this an exciting area for further research.

## Data Availability

The data supporting the findings of this study are not publicly available, as they are securely stored and fully accessible to the authors.
